# Insights and Challenges of Multi-Scale Modeling of Sarcomere Mechanics in cTn and Tm DCM Mutants—Genotype to Cellular Phenotype

**DOI:** 10.3389/fphys.2017.00151

**Published:** 2017-03-14

**Authors:** Sukriti Dewan, Kimberly J. McCabe, Michael Regnier, Andrew D. McCulloch

**Affiliations:** ^1^Departments of Bioengineering and Medicine, University of CaliforniaSan Diego, La Jolla, CA, USA; ^2^Departments of Bioengineering and Medicine, University of WashingtonSeattle, WA, USA

**Keywords:** dilated cardiomyopathy, troponin, Tropomyosin, cross-bridge, myofilament, modeling, mechanics, Markov

## Abstract

Dilated Cardiomyopathy (DCM) is a leading cause of sudden cardiac death characterized by impaired pump function and dilatation of cardiac ventricles. In this review we discuss various *in silico* approaches to elucidating the mechanisms of genetic mutations leading to DCM. The approaches covered in this review focus on bridging the spatial and temporal gaps that exist between molecular and cellular processes. Mutations in sarcomeric regulatory thin filament proteins such as the troponin complex (cTn) and Tropomyosin (Tm) have been associated with DCM. Despite the experimentally-observed myofilament measures of contractility in the case of these mutations, the mechanisms by which the underlying molecular changes and protein interactions scale up to organ failure by these mutations remains elusive. The review highlights multi-scale modeling approaches and their applicability to study the effects of sarcomeric gene mutations *in-silico*. We discuss some of the insights that can be gained from computational models of cardiac biomechanics when scaling from molecular states to cellular level.

## Introduction

Dilated Cardiomyopathy (DCM) is one of the four classified forms of cardiomyopathy besides hypertrophic cardiomyopathy (HCM), restrictive cardiomyopathy (RCM), and arrythmogenic right ventricular dysplasia/cardiomyopathy (ARVD/C) (Richardson et al., 1996; Seidman and Seidman, 2001). DCM, one of the major causes of cardiac death, is characterized by impaired systolic function and dilatation of one or both ventricles (Richardson et al., 1996; Kärkkäinen and Peuhkurinen, 2007). Hemodynamically, contractility is depressed and Pressure-Volume (PV) loops are right-shifted in DCM. In 30–50% of cases DCM is linked to familial etiology, including mutations in the regulatory thick and thin myofilament proteins - myosin, actin, the Troponin (Tn) complex, Tropomyosin (Tm), and Titin (Ttn) (Kärkkäinen and Peuhkurinen, 2007; Lu et al., 2013). Many times DCM presents with conduction defects and sequelae of other cardiac defects. This is more common with cytoskeletal and z-disc mutations (Seidman and Seidman, 2001; Chang and Potter, 2005). Genome Wide Association Studies (GWAS) have helped identify many of the sarcomeric mutations associated with the phenotype of DCM (Kamisago et al., 2000; Li et al., 2001; Olson et al., 2001; Mogensen et al., 2004; Murphy et al., 2004; Lakdawala et al., 2010, 2012a; Branishte et al., 2013; Pérez-Serra et al., 2016). Despite the identification of sarcomeric mutations associated with DCM, it is still difficult to predict the exact functional consequences of the mutation at the cellular level based on its molecular structure and function. Identified sarcomeric mutations may represent a gain of function or loss of function at the cellular level, as assessed by myofilament mechanics (Kamisago et al., 2000; Spudich and Rock, 2002; McNally et al., 2013). Furthermore, a spatial and temporal translation of the cellular level mechanical phenotype to an organ level DCM phenotype has not been seamlessly achieved. These patho-physiological translative events from genetic to molecular to cellular to organ level, underscore the need for investigation at and across all biological scales to fully comprehend the development of DCM (Spudich, 2014).

Previous experimental studies, incorporating structural data from x-ray crystallography and nuclear magnetic resonance (NMR) into molecular dynamic (MD) models and *in-vitro* molecular assays, have reported changes in molecular properties of various DCM associated sarcomeric mutants. Findings include altered calcium binding affinity (Robinson et al., 2007; Kekenes-Huskey et al., 2012), rate of cTnC hydrophobic patch opening (Dewan et al., 2016), acto-myosin affinity (Moore et al., 2012), altered surface charge distribution on coiled-coil region of Tm (Olson et al., 2001), Titin to z-disc protein T-cap/Telethonin affinity (Thirumal Kumar et al., 2016) and cTnC-cTnI interactions (Mogensen et al., 2004; Dewan et al., 2016). The impact of molecular alteration to cellular phenotype can be qualitatively or semi-quantitatively intuitive in some cases. For example a decrease in calcium binding affinity of cTnC will lead to decreased thin filament activation and force development. However, the exact quantification of such an effect is still to be reported for various mutants and unanswered questions remain. Will a 25% decrease in calcium binding affinity of cTnC lead to a 50% decrease in thin filament activation which would result in 50% decrease in maximal force response or will it trigger compensatory molecular mechanisms and result in a 10% decrease in thin filament activation and force generation? Or does a 10% decrease in thin filament activation imply a 20% decrease in calcium binding affinity of cTnC? As an example, a recent study reported that the severity of DCM is determined by the ratio of mutant to wildtype *TnnT2* gene transcript in ΔK210 cTnT transgenic mice, as absence of one allele of *TnnT2* does not lead to a protein deficit (Ahmad et al., 2008). The quantitative translation of molecular perturbation to cellular events and vice versa warrants more studies. Nonetheless, it has been reported that identified genetic mutations in contractile active force-generating sarcomeric proteins (excluding Titin) exhibit a very specific DCM phenotype without any other associated cardiac phenotype such as hypertrophy or conduction defects with a high prevalence in young people (Mogensen et al., 2004; Memo et al., 2013). This would suggest that diverse molecular abnormalities converge at the cellular phenotype, which then trigger the cardiac remodeling leading to DCM. Indeed depressed contractile function is a common cellular phenotype in DCM despite variable molecular mechanisms (Mirza et al., 2005).

Experimental studies, using the skinned muscle preparation or intact cardiomyocytes from gene-targeted mouse models (Du et al., 2007), adenoviral-mediated transfection (Morimoto et al., 2002; Lu et al., 2003; Mirza et al., 2005; Lim et al., 2008), *in-vitro* protein exchange experiments (Dweck et al., 2010) for *in-vitro* protein motility assays (Mirza et al., 2005; Memo et al., 2013), mammalian two-hybrid luciferase assay system (Mogensen et al., 2004; Murphy et al., 2004), steady-state force-calcium assays (Morimoto et al., 2002; Lu et al., 2003), and contractility assays (Biesiadecki et al., 2007; Lim et al., 2008; Dweck et al., 2010), have reported alterations in key mechanical properties of myofilaments in DCM. These include changes in calcium sensitivity (Robinson et al., 2002; Mirza et al., 2005; Du et al., 2007; Lim et al., 2008; Dweck et al., 2010; Memo et al., 2013; Kalyva et al., 2014), thick and thin myofilament cooperativity and cross-bridge (XB) cycling rates (Moore et al., 2012), as a functional outcome of many identified sarcomeric DCM mutations. Most studies have reported a decrease in calcium sensitivity of myofilaments as a consistent functional phenotype for DCM identified sarcomere mutations (Murphy et al., 2004; Mirza et al., 2005; Du et al., 2007; Robinson et al., 2007; Memo et al., 2013). Interestingly, I61Q cTnC mutant in mice (neither found nor associated with DCM clinically), that has decreased calcium myofilament binding affinity, recapitulates DCM phenotype cellularly and *in vivo* (Davis et al., 2016). However, exceptions to this have been reported wherein both an increase and no change in calcium sensitivity were reported in DCM (Dweck et al., 2008; Memo et al., 2013). Dweck et al. reported that there is a decrease in calcium sensitivity of tension development in G159D cTnC mutant only when it is incorporated in regulated actomyosin filaments and not in isolated cTnC (Dweck et al., 2008), highlighting the effect of protein interactions as the hierarchy of structural organization becomes more physiological and complex in the contractile apparatus. Furthermore, Memo et al. reported a decrease in calcium sensitivity of myofilaments for four DCM associated mutants (K36Q TnI, R141W TnT, ΔK210 TnT, E40K Tm), no change in calcium sensitivity of one mutant (E54K Tm) and increase in calcium sensitivity in another (D230N Tm) (Memo et al., 2013). These observations suggest that decreased calcium sensitivity is a dominant stimulus sufficient to cause DCM, but is neither necessary, nor the only cellular mechanism triggering the remodeling observed in DCM. A recent study postulated that the blunting of the relationship between calcium sensitivity of myofilaments and PKA mediated beta-adrenergic stimulation via cTnI phosphorylation (Memo et al., 2013) in sarcomeric DCM mutants might be the defining cellular phenotype for DCM regardless of the directional shift in calcium sensitivity. It should be noted that aside from DCM mutations in contractile sarcomere proteins that lead to diminished force production, mutations in other key cytoskeletal and sarcomere proteins like the z-disc proteins and Titin can lead to disruptions in transmission of force, sensing of force and mechano-transduction which are also causative toward DCM (Chang and Potter, 2005). These observations suggest that molecular interactions and effects of various DCM mutants converge to a depressed contractile phenotype at the cellular level due to alterations in (a) calcium sensitivity of myofilaments, (b) thin-filament activation, (c) maximal ATPase activity, (d) *in-vitro* motility (e) calcium affinity of Tn and (f) mechano-transduction, thereby triggering the signaling mechanism leading to DCM (Chang and Potter, 2005; Lakdawala et al., 2012b). Further studies are warranted to establish the key converging cellular mechanism/s and signaling pathway/s in DCM.

It is interesting to note that both HCM and DCM can be caused by different mutations within the same contractile protein gene. HCM is a more common familial disease with a contrasting phenotype of hypertrophied ventricle and preserved systolic function. These observations imply that there are either two different signaling pathways distinguishing these phenotypes or a graded response within the same pathway. An earlier study in transgenic mice with a truncation allele of Myosin Binding protein C (MyBP-C), known to cause HCM in man, exhibited a graded response such that heterozygotes resulted in HCM and homozygotes in DCM (McConnell et al., 1999). In contrast, Ahmad et al. and Ramratnam et al. reported that the ratio of mutated to wildtype transcript of TnT is critical in determining severity of DCM and not haploinsufficiency (Ahmad et al., 2008; Ramratnam et al., 2016). Nonetheless, given that most studies report a contrasting cellular phenotype to HCM with directionally opposite shifts in calcium sensitivity, two separate pathway theory is strongly supported. This is corroborated by a recent study where the authors propose that modeling the tension integral of cardiomyocytes can help distinguish between DCM and HCM (Davis et al., 2016). Notably, a recent RNAseq study profiling molecular signaling in DCM and HCM reported that profibrotic and metabolic networks can distinguish between the two phenotypes (Burke et al., 2016). Multi-scale studies quantifying genotype to functional cellular phenotype will help address these theories. The convergence of various molecular defects to fewer cellular phenotypes to a singular DCM phenotype via cardiac remodeling is notable.

Clinically, mutations are prevalent from birth in familial DCM. However, the temporal transition to DCM is not yet understood (Tardiff, 2012). A recent study using tissue Doppler and strain echocardiography, showed that even early on there are subtle indications (Lakdawala et al., 2012b). In subclinical DCM mutation carriers, reduced systolic myocardial velocity, strain and strain-rate were reported despite normal LV geometry, ejection fraction and diastolic function (Lakdawala et al., 2012b). Early diagnosis in such cases can provide with much needed time (Lakdawala et al., 2012b). It is also important to note that some HCM patients develop dilated ventricles at later stages for example in patients with mutations E180V in Tm and R92W in cTnT (Chang and Potter, 2005). Studies have shown this to be distinct from DCM phenotype (Ohba et al., 2007). So in familial DCM at the whole heart level there is subtle manifestation of DCM from birth and transition to late stage DCM eventually. Mechanical and pathway driven changes lead to this cardiac remodeling (Burke et al., 2016; Davis et al., 2016). Investigations on the effects of DCM mutations quantitatively and qualitatively, in animal model studies, linking genotype to muscle phenotype i.e., from molecular level changes to myofilament level changes to intact tissue level changes to whole heart level changes are scarce (Ahmad et al., 2008; Ramratnam et al., 2016). This is due to the technical challenges and expense of carrying out such an expansive study to experimentally measure data on a single DCM mutation. Additionally, as studies report cellular data, isolating the singular effect of the genetic mutation becomes difficult due to downstream effects of the mutation that also contribute to the systemic perturbations leading to the phenotype.

A potential way to study the mechanical effects of genetic mutations comprehensively is by multi-scale computational modeling. Cardiac muscle biomechanics has been experimentally and computationally investigated in molecular machineries of sarcomeric protein complexes (Varguhese and Li, 2011; Lindert et al., 2012b, 2015), in thick and thin myofilaments (Rice and de Tombe, 2004; Campbell et al., 2010; Dewan et al., 2016), in isolated cardiomyocytes under steady-state and dynamic conditions (Hussan et al., 2006; Rice et al., 2008), in myocardium, and in the whole heart (Göktepe et al., 2010; Campbell and McCulloch, 2011; Trayanova, 2011; Kerckhoffs et al., 2012; Zhang et al., 2016). An integrative mathematical formulation to scale from protein level changes to the cardiomyocyte function is yet to be implemented. Parameterization of computational models requires collating data from various studies for a single mutation at various spatial and temporal scales. This results in data from variable experimental conditions and requires standardization in terms of (a) temperature, (b) species, and (c) bridging of spatial and temporal scales, in order to input model parameters (Tøndel et al., 2015; Dewan et al., 2016). In this review we discuss various *in silico* approaches and the challenges thereof, by examining previously identified and experimentally studied DCM mutations in thin filament proteins Tn and Tm, with the goal of elucidating the mechanical effects of sarcomeric mutations from molecular level to the cellular scales.

## Cardiac muscle contraction and effects of DCM mutants in regulatory thin filament proteins cTn and Tm

Cardiac muscle contraction is triggered following calcium induced calcium release (CICR) from the sarcoplasmic reticulum (SR) (Fabiato, 1983). In 1954, two groundbreaking studies proposed the sliding filament theory of muscle contraction describing the molecular basis of muscle contraction (Huxley and Niedergerke, 1954; Huxley and Hanson, 1954). According to the sliding filament theory, thin (actin) and thick (myosin) filaments slide past each other, while maintaining absolute lengths, during contraction to generate contractile force. Cardiac TnC (cTnC), a 18 kDa thin filament protein within the Tn complex, is a key regulator of muscle contraction. Calcium binding to cTnC triggers the biomechanical cascade of contraction events within the sarcomere (Gordon et al., 2000). Calcium binding to cTnC induces a conformational change within the Tn complex, thereby displacing Tropomyosin (Tm) from actin filaments to expose myosin binding sites and increasing the probability of cross-bridges cycling. In the resting state of the myofilaments when Ca^2+^ is not bound to cTnC, cTn complex anchors Tm in a “Blocked” position, thereby, sterically hindering access to sites on the thin filament where Myosin S1 heads can bind to actin to form XBs (Gordon et al., 2000). Upon Ca^2+^ binding cTnC undergoes a conformational change, which exposes a hydrophobic patch within cTnC and allows cTnC to bind to the switch peptide subunit of cardiac Troponin I (cTnI). Once the cTnC has bound to the cTnI switch peptide, cTn complex releases the anchoring Tm, allowing the Tm molecule to slide around the actin filament. Tm molecules overlap, which leads to cooperative interactions between nearest-neighbor thick-thin myofilament proteins that can be affected by the stiffness of the Tm molecule. Tm moves from the Blocked state to a Closed state in which myosin binding sites are partially exposed, and then to an Open state where myosin such that actin binding sites on the thin filament are exposed, thus initiating XB cycling (Vibert et al., 1997; Gordon et al., 2000; de Tombe et al., 2010).

**Figure 1:**
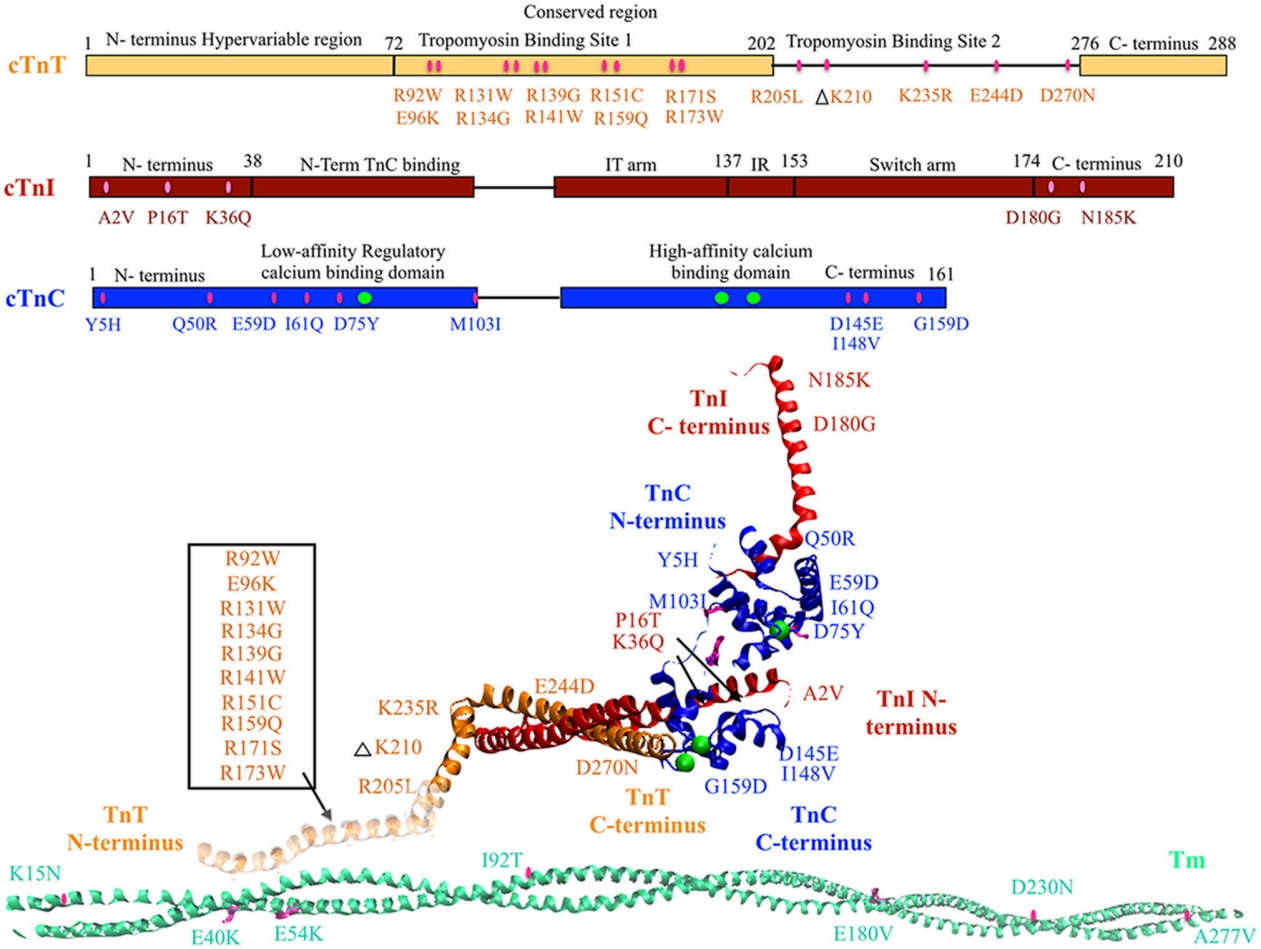
**DCM associated mutations shown in regulatory thin filament proteins cTn and Tm**. DCM mutations for cTn are shown in the cartoon representing primary amino acid sequence of cTnC, cTnI, and cTnT and in the cTnC crystal structure of the 52 kDa domain of human cTn (PDB ID—1J1E) in the calcium saturated form (cTnC in blue, cTnI in red, cTnT in orange, calcium in green, mutations in pink). Residues 1–182 are missing in the crystal structure of cTnT—the mutations in that region have been enclosed within an open box and point to a cartoon rendering of residues 1–182 of cTnT (faded orange ribbon structure juxtaposing Tm). DCM mutations in Tm are shown in crystal structure for Tm (PDB ID—1C1G) (Tm in teal, mutations in pink).

Mutations in regulatory thin filament sarcomeric proteins Tn and α-Tm have been associated with DCM (Table 1) (Figure 1). A clinical study of idiopathic DCM patients found mutations in the Tn complex in 7% of patients, with severe prognosis for patients with mutations in cTnC (Chang and Potter, 2005; Lu et al., 2013). cTnC D75Y/E59D missense mutation was detected in an adult male who suffered sudden cardiac death as a result of idiopathic DCM (Lim et al., 2008). D75Y and E59D are point mutations located in the low affinity Ca^2+^ binding site on cTnC near the N-terminus. Studies in skinned and intact cardiomyocytes have reported a marked decrease in calcium sensitivity, cell shortening and force production for the double mutant (D75Y/E59D) and D75Y alone in spite of the fact that the mutations do not influence the intracellular calcium homeostasis (Lim et al., 2008; Dweck et al., 2010). However, both D75Y and E59D are required to reduce the actomyosin ATPase activity and maximal force in muscle fibers, indicating that E59D enhances the effects of D75Y (Dweck et al., 2010). In addition to D75Y and E59D, the G159D cTnC mutation has been found in human DCM patients and has been shown to impair cTnC-cTnI interaction and decrease Ca^2+^ binding affinity (Mogensen et al., 2004; Biesiadecki et al., 2007; Robinson et al., 2007; Baryshnikova et al., 2008). G159D cTnC mutant exhibits reduced opening and closing rates of N-terminus of cTnC post-calcium binding and dissociating respectively (Dong et al., 2008). Additionally, G159D cTnC mutant abolishes the accelerated closing rate of the N-terminus of cTnC triggered by PKA mediated phosphorylation of cTnI (Dong et al., 2008). Four rare clinical variates (Y5H, M103I, D145E, and I148V) of *TnnC1* have been reported in association with DCM (Hershberger et al., 2010), of which Y5H cTnC mutation was reported in a pediatric patient with idiopathic DCM concomitant with a mutation in Myosin (Rampersaud et al., 2011). Three of these (Y5H, M103I, and I148V) showed decreased calcium sensitivity of myofilaments and impaired response of the myofilament to undergo Ca^2+^ desensitization upon PKA phosphorylation (Pinto et al., 2011). The fourth variant D145E presented with a MyBP-C rare variant. Given that D145E mutation shows increased calcium sensitivity and is associated with HCM, it is quite plausible that the concomitantly present MyBP-C mutation mediated the observed DCM response (Landstrom et al., 2008; Pinto et al., 2011). Additionally, cTnC mutant Q50R has been identified in a DCM family with a member with the rare disease of peripartum dilated cardiomyopathy (van Spaendonck-Zwarts et al., 2010).

**Table 1:** **DCM associated cTn and Tm mutants and their known molecular (M) effects, cellular (C) effects, and organ level (O) effects**.

**Protein**	**Mutation**	**Effects at Molecular (M), Cellular (C), and Organ (O) level**
cTnC	D75Y	M: Decreased calcium binding affinity of TnC, decreased free energy of cTnC conformational change (hydrophobic patch opening) on calcium binding (Dewan et al., 2016)
		C: Decreased calcium sensitivity of myofilaments, decreased cell shortening, decreased force production (Lim et al., 2008; Dweck et al., 2010)
	E59D	M: Decreased calcium binding affinity of TnC, increased free energy of cTnC conformational change (hydrophobic patch opening) on calcium binding (Dewan et al., 2016)
		C: No apparent change in calcium sensitivity, no known changes relative to wildtype (Lim et al., 2008; Dweck et al., 2010)
	E59D/D75Y	C: Reduced maximum myofibrillar ATPase activity and decreased maximum force of contraction in skinned fibers (Dweck et al., 2010)
	G159D	M: Decreased calcium binding, reduced opening and closing rate sof cTnC N-terminus, increased free energy of cTnC conformational change (hydrophobic patch opening) on calcium binding, Impaired cTnC-cTnT interaction, blunted cTnC-cTnI interaction effect based on PKA phosphorylation, impaired anchoring of cTnC to cTnI (Mogensen et al., 2004; Robinson et al., 2007; Baryshnikova et al., 2008; Dong et al., 2008; Dewan et al., 2016)
		C: Decreased calcium sensitivity of myofilaments, decreased maximal ATPase activity and myofilament sliding speed, dissociation between calcium sensitivity and PKA mediated beta adrenergic response to TnI phosphorylation (Mirza et al., 2005; Biesiadecki et al., 2007; Robinson et al., 2007; Memo et al., 2013)
	Y5H	C: Decreased calcium sensitivity of myofilaments, dissociation between calcium sensitivity and PKA mediated beta adrenergic response to TnI phosphorylation, diminished capacity to recover force in skinned fibers, impaired thin-filament activation in ATPase assays (Pinto et al., 2011)
	M103I	C: Decreased calcium sensitivity of myofilaments, complete dissociation between calcium sensitivity and PKA mediated beta adrenergic response to TnI phosphorylation, diminished capacity to recover force in skinned fibers, impaired thin-filament activation in ATPase assays (Pinto et al., 2011)
	D145E	C: Increased calcium sensitivity of myofilaments, previously associated with HCM, reported with rare mutation in MyBP-C (Pinto et al., 2011)
	I148V	C: Decreased calcium sensitivity of myofilaments, dissociation between calcium sensitivity and PKA mediated beta adrenergic response to TnI phosphorylation, diminished capacity to recover force in skinned fibers, impaired thin-filament activation in ATPase assays (Pinto et al., 2011)
	Q50R	Known to occur in peripartum DCM (van Spaendonck-Zwarts et al., 2010)
cTnI	A2V	M: Impaired cTnC-cTnI interaction (Murphy et al., 2004)
	K36Q	M: Decreased calcium binding to cTnC, Mediate movement of N-terminus region of cTnI upon phosphorylation of S22/23 by PKA (Howarth et al., 2007)
		C: Decreased maximal ATPase activity, decreased calcium sensitivity of actin-myosin S1 ATPase, dissociation between calcium sensitivity and PKA mediated beta adrenergic response to TnI phosphorylation (Carballo et al., 2009; Memo et al., 2013)
	D180G	Associated with a pediatric patient with familial DCM (Rampersaud et al., 2011)
	N185K	C: Decreased maximal ATPase activity, decreased calcium sensitivity of actin-myosin S1 ATPase (Carballo et al., 2009)
	P16T	Associated with DCM (Murakami et al., 2010)
cTnT	E96K	Associated with idiopathic DCM; reported in 5mo patient (Rampersaud et al., 2011)
	R131W	M: Enhanced cTnC-cTnI and impaired cTnC-cTnT interactions (Mogensen et al., 2004)
		C: Decreased calcium sensitivity, decreased maximal ATPase activity and myofilament sliding speed (Mirza et al., 2005)
	R134G	C: Decreased calcium sensitivity of force development (Hershberger et al., 2009; Rampersaud et al., 2011)
	R139H	C: Decreased calcium sensitivity of force development; late onset DCM (Morales et al., 2010)
	R141W	M: Increased affinity of cTnT to Tm (Lu et al., 2003)
		C: No change (Venkatraman et al., 2003)/Decreased calcium sensitivity of myofilaments, decreased maximal ATPase activity and myofilament sliding speed, dissociation between calcium sensitivity and PKA mediated beta adrenergic response to TnI phosphorylation, increases in diastolic and peak intracellular calcium (Mirza et al., 2005; Memo et al., 2013; Ramratnam et al., 2016)
		O: Dose-dependent mutation effect, DCM phenotype, slower intrinsic rates in sinus rhythm, reduced peak heart rate in response to isoproterenol (Ramratnam et al., 2016)
	R151C	C: Decreased calcium sensitivity of force development (Hershberger et al., 2009)
	R159Q	C: Decreased calcium sensitivity of force development (Hershberger et al., 2009)
	A171S	Gender dependent DCM, more severe effects in males (Stefanelli et al., 2004)
	R173W	C: Altered calcium regulation and lower contractility (Sun et al., 2012; Davis et al., 2016)
	R205W	C: Decreased calcium sensitivity of force development (Hershberger et al., 2009; Rampersaud et al., 2011)
	R205L	M: Impaired cTnC-cTnI and cTnC-cTnT interactions (Mogensen et al., 2004)
		C: Decreased calcium sensitivity of myofilaments, decreased maximal ATPase activity and myofilament sliding speed (Mirza et al., 2005)
	ΔK210	M: Impaired cTnC-cTnI and cTnC-cTnT interactions (Mogensen et al., 2004)
		C: Altered calcium sensitivity of myofilaments, decreased maximal ATPase activity and myofilament sliding speed (Morimoto et al., 2002; Robinson et al., 2002, 2007; Venkatraman et al., 2003; Mirza et al., 2005; Du et al., 2007; Ahmad et al., 2008)
		O: Dose-dependent mutation effect, DCM phenotype (Ahmad et al., 2008)
	K235R	M: Impaired cTnC-cTnI and cTnC-cTnT interactions (Mogensen et al., 2004)
	E244D	Identified in DCM associated proband and pediatric patient with familial DCM; previously associated with HCM (Hershberger et al., 2009; Rampersaud et al., 2011)
	D270N	M: Impaired cTnC-cTnI and cTnC-cTnT interactions (Mogensen et al., 2004)
		C: Decreased calcium sensitivity of myofilaments, decreased maximal ATPase activity and myofilament sliding speed, decreased cooperativity (Mirza et al., 2005; Robinson et al., 2007)
αTm	E40K	M: Destabilization of Tm Dimers, may alter Tm-actin interaction (Olson et al., 2001; Chang et al., 2014)
		C: Decreased calcium sensitivity of myofilaments, decreased maximal ATPase activity and myofilament sliding speed, dissociation between calcium sensitivity and PKA mediated beta adrenergic response to TnI phosphorylation (Mirza et al., 2005; Memo et al., 2013)
	E54K	M: Destabilization of Tm Dimers, increased stiffness and decreased curvature in Tm (Chang et al., 2014; Zheng et al., 2016)
		C: Decreased calcium sensitivity of myofilaments, no effect on maximal ATPase activity or myofilament sliding speed, dissociation between calcium sensitivity and PKA mediated beta adrenergic response to TnI phosphorylation (Mirza et al., 2005; Memo et al., 2013)
	D230N	C: Decreased calcium sensitivity of myofilaments, dissociation between calcium sensitivity and PKA mediated beta adrenergic response to TnI phosphorylation (Lakdawala et al., 2010; Memo et al., 2013)
	K15N	Associated with pediatric patients with familial DCM (Rampersaud et al., 2011)
	I92T	Associated with pediatric patient with familial DCM (Rampersaud et al., 2011)
	A277V	Associated with pediatric patient with familial DCM (Rampersaud et al., 2011)

A DCM associated mutation in cTnI, A2V, was found to hinder cTnC-cTnI interaction via mammalian two-hybrid luciferase assay (Murphy et al., 2004). Two other cTnI mutants, K36Q, and N185K, were reported in 2009 and found to decrease calcium sensitivity of actin-myosin S1 ATPase, maximal ATPase activity and reduce calcium binding affinity of cTnC (Carballo et al., 2009; Lu et al., 2013). Two more cTnI mutants P16T and D180G were reported recently (Murakami et al., 2010; Rampersaud et al., 2011).

An earlier study employing two-hybrid assays to investigate cTnT mutations R131W, R205L, and D270N, all of which have been found in human DCM patients, impaired cTnC-cTnI/cTnC-cTnT interaction (Mogensen et al., 2004). A contractility study in rabbit muscle fibers reported that these mutations and TnT R141W decrease Ca^2+^ sensitivity of force generation, maximal ATPase activity and myofilament sliding speed (Mirza et al., 2005). Additionally, Robinson et al. reported a decrease in calcium sensitivity of these cTnT mutants as well with the exception of cTnT mutation D270N. D270N cTnT led to a decrease cooperativity of Ca^2+^ binding but not overall Ca^2+^ affinity of cTn as measured by pCa50 in reconstituted thin filaments (Robinson et al., 2007). The ΔK210 cTnT mutation has been shown to decrease Ca^2+^ sensitivity of force generation and hinder cTnT-cTnI interaction without affecting maximum force generation (Morimoto et al., 2002; Mogensen et al., 2004; Du et al., 2007; Robinson et al., 2007). There are conflicting reports on the effect of ΔK210 on cooperativity of force generation (Venkatraman et al., 2003; Du et al., 2007; Robinson et al., 2007). Four missense mutations, R134G, R151C, R159Q, and R205W, in cTnT were identified in probands with familial DCM (Hershberger et al., 2009), of which two (R134G, and R205W) were also reported in pediatric patients with familial DCM (Rampersaud et al., 2011). Functional analysis of these mutations in reconstituted myocytes showed decreased calcium sensitivity of force development (Hershberger et al., 2009). Additionally, a HCM associated mutant E244D, was identified in a DCM associated proband (Hershberger et al., 2009) and in a pediatric patient with familial DCM (Rampersaud et al., 2011). Further, a cTnT mutation R139H showed decreased calcium sensitivity and was reported in late onset DCM in a 70 year old woman (Morales et al., 2010). This is interesting as usually cTnT mutations are associated with early onset and aggressive form of DCM. Lastly, E96K cTnT mutation was reported in a 5 month patient with idiopathic DCM (Rampersaud et al., 2011).

Tm mutations E40K and E54K were identified in a GWAS, and when reconstituted in rabbit muscle fibers were found to decrease Ca^2+^ sensitivity (Olson et al., 2001; Mirza et al., 2005). Interestingly, the E40K mutation was found to decrease myofilament sliding speed while the E54K mutation had no effect on sliding speed (Mirza et al., 2005). Both mutations caused localized destabilization of the Tm dimers and affect interactions with actin which would then directly affect ATPase activity (Chang et al., 2014). In addition, D230N Tm mutation showed decreased calcium sensitivity of myofilaments, and dissociation between calcium sensitivity and PKA mediated beta adrenergic response to TnI phosphorylation (Lakdawala et al., 2010; Memo et al., 2013). Lastly, multiple Tm mutations (K15N, I92T, A277V) have been reported in pediatric cases with idiopathic or familial DCM (Rampersaud et al., 2011).

The above-mentioned familial mutations in the regulatory proteins cTn and α-Tm, that have been identified in human patients over the course of years, display some wide-ranging molecular effects that mainly converge to few cellular mechanisms such as altered calcium sensitivity and decreased ATPase activity ultimately leading to depressed contractile force observed in DCM. A recent innovative motility assay study proposed that the varying experimental findings of DCM-associated thin filament mutations in α-Tm and cTn can be explained by a decoupling of Ca^2+^ sensitivity from cTnI phosphorylation by PKA (Memo et al., 2013). This finding, along with the wide variety of mutations in thin filament proteins that have been connected to the development of DCM, underscores the urgency of studying the complicated cascade of contraction events as a whole in order to create a cohesive picture of DCM causes and progression. Genetically engineered animal models provide an opportunity to understand the sequelae of molecular and cellular events as they translate to the whole heart level. Notably, genetically engineered mice with sarcomeric mutations ΔK210 (Du et al., 2007; Ahmad et al., 2008) and R141W (Ramratnam et al., 2016) in cTnT have been generated. These studies showed a gene-dosage effect on the cardiac phenotype and recapitulated the human phenotype. Despite the key genotype-to-phenotype insights gleaned from animal model studies, most experimental studies have so far been conducted in reconstituted *in vitro* systems. Majority of experimental studies have measured the effects of thin filament protein mutations on cell-level function, but have not looked more closely into molecular mechanisms or expression of dysfunction on the whole heart level. Multi-scale computational modeling offers a complementary set of tools that can help us understand the spatial and temporal transition to clinical DCM.

## Insights and challenges from molecular modeling of regulatory thin filament protein mutations

Structural determination from x-ray crystallography and NMR studies has helped provide key insights into molecular basis of cTnC function (Li and Hwang, 2015). Previously published experimental and theoretical studies have used these structural data to probe rapid, nanosecond and microsecond timescale conformational dynamics, by using Brownian Dynamics (BD) and Molecular Dynamics (MD) simulations, that are correlated with calcium binding (Kekenes-Huskey et al., 2012; Kalyva et al., 2014). MD and BD simulations are useful *in-silico* approaches that have been employed to understand the molecular basis of altered structural and functional dynamics of regulatory myofilament proteins such as cTnC in varied states. For example, Varughese and Li investigated, with MD, changes in the structural dynamics of cardiac Tn, including TnC, upon binding bepridil, a known inotropic agent (Varguhese and Li, 2011; Lindert et al., 2012b) combined long time-scale MD simulations and BD simulations to understand the dynamics of wild-type TnC in its calcium-free, calcium-bound, and TnI-bound states, as well as V44Q (Lindert et al., 2012a).

Intra-molecular dynamic changes in cTn can cause alterations in: (a) the calcium binding affinity of cTnC; (b) the rate of calcium dissociation from cTnC; (c) the forward rate of cTnC conformation transition; (d) the reverse rate of cTnC conformation transition; and (e) the structure of cTnC such that there are differences in charge within the exposed hydrophobic patch (Dewan et al., 2016). Additionally, cTn mutations can affect interactions between cTnC, cTnI, and cTnT as well as downstream contractile proteins such as Tm and actin (Mogensen et al., 2004; Robinson et al., 2007). A recent *in silico* study (Dewan et al., 2016) employed MD and BD simulations to postulate the decreased calcium binding affinity of cTnC and the altered rate of hydrophobic patch opening within cTnC as the molecular basis of the reported changes in calcium sensitivity and force production in the D75Y cTnC DCM mutant. An MD study on residues 70–110 of cTnT found that familial HCM -linked mutations R92L and R92W, located near the Tm binding domain, lead to increased hinge movement downstream on the cTnT molecule as well as decreased helical stability (Ertz-Berger et al., 2005). Importantly, the study found that divergent phenotypes emerge in a live mouse model of these mutations, which demonstrates a limitation to modeling isolated proteins (Ertz-Berger et al., 2005).

MD simulations have the potential to uncover biophysical effects of DCM associated mutations in cardiac Tm. Recent MD studies have explored the properties of Tm in healthy cases as well as patho-physiological cases, noting that Tm stiffness may impact downstream contractility events greatly (Li et al., 2010a,b; Loong et al., 2012; Lehman et al., 2015). A 2012 study used MD to study familial HCM associated Tm mutations D175N and E180G and found that both mutations lead to increased flexibility of Tm and therefore, decreased persistence length of the molecule (Li et al., 2012). Another MD study has also been performed on HCM Tm mutations E62Q, A63V, K70T, V95A, D175N, E180G, L185R, E192K in order to explore the effects of point mutations on Tm flexibility and Tm-actin interactions (Zheng et al., 2016). In the case of DCM, a time-independent electrostatic snapshot of the DCM associated Tm mutation showed that E54K and E40K mutations alter the surface charge of Tm, which may affect Tm-actin interaction (Olson et al., 2001; Chang et al., 2014). A time-dependent MD study was also performed on the E54K Tm mutant in combination with 7 actin monomers and showed that this mutation causes increased stiffness and decreased curvature in the Tm molecule overall while stabilizing and destabilizing the coiled coil structure in different regions of the molecule and greatly weakening Tm-actin binding (Zheng et al., 2016). More MD studies are needed in the area of Tm DCM mutations in order to visualize effects of other mutations, such as E40K, that may affect Tm-actin and Tm-cTn interactions.

*In-silico* investigation of sarcomere protein structure-function dynamics by MD and BD simulations provides key insights toward understanding molecular effects of genetic mutations and post-translational modifications (Lindert et al., 2015; Dewan et al., 2016). However, these structure-function dynamics can change significantly when myofilament proteins interact with one another in an integrated physiological system. It has been reported that kinetic rates of a given state transition vary in isolated molecular states and integrated myofilament states (Davis and Tikunova, 2008). This was well demonstrated in a previous study where the off-rates of calcium binding were studied in depth from isolated cTnC molecule to a structurally integrated myofilament preparation (Davis and Tikunova, 2008). The whole cTn complex has been modeled using MD, both with and without Ca^2+^ bound (Varughese et al., 2010; Jayasundar et al., 2014). A 2014 study proved the value of modeling the complex as a whole, because removing Ca^2+^ from the regulatory binding pocket on cTnC affected cTnC hydrophobic patch opening as well as the folding and flexibility of the cTnI switch region (Jayasundar et al., 2014). Moving further up in scale, an integrated MD study of a whole thin filament including cTn, 14 actin monomers, and two overlapping Tm molecules illustrates the importance of modeling inter-protein interactions along the thin filament (Manning et al., 2011). The MD simulation performed was only 1 ns in length, but was able to accurately capture the rotation of the I-T arm in the Tn complex as a direct consequence of Ca^2+^ binding (Manning et al., 2011). The model was used to study cTnT mutations R92W and R92L associated with familial HCM and found that both mutations decrease bending forces in the hinge region of cTnT which affects Tm interaction, a downstream effect that may not have been captured if the study were performed on isolated cTnT (Manning et al., 2012).

Integration of molecular level state-transition kinetic data into the sarcomere level is a key challenge yet to be solved for using *in-silico* methods. At the molecular level it takes about 5–10 μs for calcium to bind to cTnC (Lindert et al., 2012a,b). With the state-of-the-art supercomputers we are now able to simulate molecular events such as calcium-binding events and conformational state-transitions within cTnC (a relatively small protein–18 kDa), events that occur in the microseconds range. At the myofilament level it takes about 700 ms for a contraction-relaxation cycle to take place in a sarcomere (Kerckhoffs et al., 2010; Tøndel et al., 2015). Recording the kinetics of all inter-molecular and intra-molecular state transitions for every myofilament protein during one contraction-relaxation cycle in a sarcomere is a tremendous task, as illustrated by the 1 ns upper limit of an MD simulation incorporating the full thin filament (Manning et al., 2011). This is due to the enormous computational power and speed that would be needed to solve for longer time scale simulations and bulky proteins, such as Titin (3.9 MDa), Myosin (220 kDa), and Tropomyosin (37 kDa), that form the backbone of the sarcomeric thin and thick myofilament protein complexes. Nevertheless, key insights into molecular behavior of bulky proteins like Titin that are commonly known to be mutated in DCM have been achieved by MD simulations (Herman et al., 2012). An earlier MD study of Titin wherein, single Ig domains of Titin were stretched reported sequential unfolding of Ig domains corroborating experiments (Lu et al., 1998, 2000; Gao et al., 2002). A recent study of Titin examined the hydrophobic core region of the protein associated with a DCM mutation V54M and reported destabilization of transition from bend to coil in secondary structure of Titin and reduced affinity to Z-disc protein T-cap/telethonin (Thirumal Kumar et al., 2016). Given that Titin mutations are commonly associated with DCM, molecular modeling of Titin domains associated with DCM is mandated. While we have much to gain from MD simulation studies, gaps in our experimental knowledge toward understanding dynamics of molecular level interactions and kinetics of state transitions between thick and thin myofilament proteins compound the challenges in standardizing conditions and validating results from the molecular simulation studies.

## Insights and challenges from *In-silico* translation of molecular level changes to thin filament mechanics

The mathematical formulation of cardiac myofilament models that explicitly incorporate spatio-temporal acto-myosin interactions and stochastic XB formation to compute contractile force have lagged behind electrophysiological models of the heart (Rice and de Tombe, 2004; Zhang et al., 2016). This is largely due to the (a) paucity of kinetic data on thick-thin myofilament interactions and molecular state-transitions, (b) requirement of partial-differential equations (PDEs) to solve for explicit spatio-temporal acto-myosin interactions, (c) lack of complete understanding of translation of steady-state contractile force into a length and load-dependent dynamic contractile force response via XB cycling, (d) difficulty in solving for computationally expensive stochastic interactions, (e) partial understanding of cooperative mechanisms involved in myofilament activation, and (f) technical gaps in our knowledge due to species differences and varied experimental conditions in scientific studies. Nonetheless, ordinary differential equation (ODE) and Monte Carlo Markov models of regulated co-operative myofilament activation with nearest neighbor interactions, wherein some molecular states are lumped together empirically and model parameters are optimized such that the best-fit of the base model to the experimentally measured steady-state force-calcium data-sets is achieved, have been formulated (Noble, 2002; Rice et al., 2003, 2008; Puglisi et al., 2004; Rice and de Tombe, 2004; Hussan et al., 2006; Campbell et al., 2010; Aboelkassem et al., 2015; Sewanan et al., 2016). These relatively simplified mean-field Markov models of cooperative myofilament activation and contraction have helped provide deeper insights as to how changes in inter-molecular and intra-molecular interactions in myofilament proteins can alter steady-state myofilament properties (myofilament calcium sensitivity and cooperativity of myofilament activation) and function (maximal force generation), which can then translate to cardiac pump dysfunction as reported in DCM.

Arguably the most logical type of sarcomere-level model to begin with, when testing the effects of point mutations on thin filament proteins, is a Markov model of thin filament activation. One such model, originally developed in 2010 and since expanded for a variety of applications, consists of 26 spatially explicit regulatory units (RUs), each RU including 7 actin monomers, one Tm molecule, and cTn (Campbell et al., 2010). The model captures cooperativity of thin filament activation by relying on the Tm position (blocked, closed, or open) of each RU's nearest neighbor in order to determine rates governing state transitions. Using this model as a starting point, further studies have been able to test a variety of contractility protein mutations at a larger spatial and temporal scale than MD simulation can reasonably accomplish. A relevant example is a recent study that simulated Tm mutations E180G and D175N, which have been found in HCM patients, using a Monte Carlo framework extension of the Campbell model (Sewanan et al., 2016). MD simulations indicated that both Tm mutation increase Tm flexibility while lowering persistence length of the molecule (Li et al., 2012). The thin filament model was able to simulate experimentally gathered wildtype and mutant contraction data by altering Tm persistence length, transition rates between the blocked and closed Tm states, and percentage of the XB cycle spent in the attached force-producing state (Sewanan et al., 2016). MD simulation data of isolated Tm can offer information on persistence length but not on Tm-actin interaction or XB cycling, which were discovered to be possible downstream effects of these mutations.

**Figure 2:**
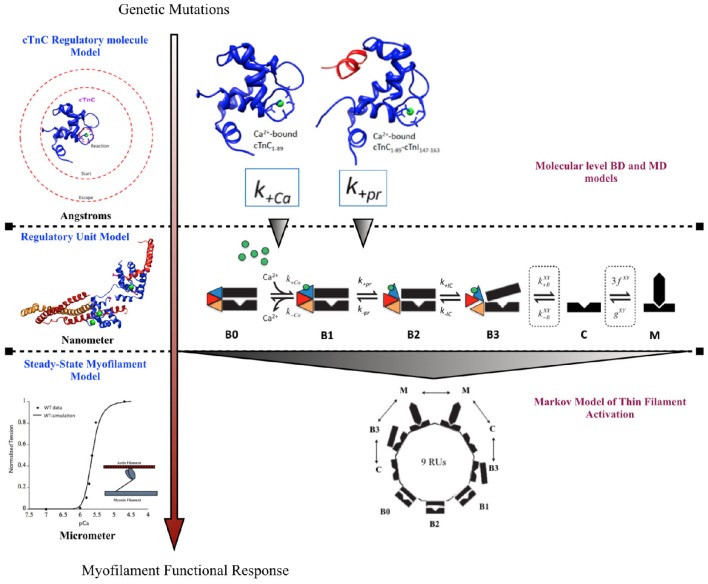
**Schematic showing incorporation of molecular data of cTnC activation obtained from BD and MD simulations into a six-state Markov model of myofilament activation at steady-state conditions, as an example of bridging molecular to cellular scales in a modeling study by Dewan et al. (2016)**.

A previously published multi-scale modeling study from our group was one of the first studies to directly incorporate the molecular changes computed from BD and MD simulations, in the DCM mutant D75YcTnC, into a six-state Markov model of steady-state myofilament contraction (Dewan et al., 2016) (Figure 2). The key results from the study reported that the intra-molecular changes (decreased calcium binding affinity of cTnC and depressed rate of hydrophobic patch opening during cTnC conformational change) in D75Y cTnC mutant are sufficient to explain the observed decrease in myofilament calcium sensitivity under steady-state experimental conditions in skinned cardiomyocytes. Additionally, the study highlighted how DCM mutants like E59D cTnC which appear to have healthy myofilament response in skinned myofilament experiments, can in fact be harboring molecular modifications which can potentially turn deleterious under stressful conditions for the myocardium. A weakness of this study was that they did not model the effects of the double mutant D75Y/E59D. This would have been more appropriate as both mutations, D75Y, and E59D are needed for a reduction in maximum myofibrillar ATPase and maximum force of contraction in skinned fibers. Additionally, The D75Y mutant is important for the effect seen in calcium binding affinity, however the “cellular” phenotype may not be explained solely by a single mutation based on the *in vitro* data. This study was instrumental in its scope, as it bridged the genotypic defects at sarcomeric protein level to myofilament phenotype *in silico*, by directly incorporating molecular parameters from BD and MD simulations into appropriate markov states of cTnC activation. The study again displays the numerous challenges for the mathematical modeler in scaling from molecular level to myofilament levels to compute steady-state contractile force, as MD data was insufficient to fully capture cellular mutant contractility changes which may be due to cTnC-cTnI interaction or other protein interactions in the sarcomere as shown in case of G159D cTnC mutant. It is important to note that prediction of steady-state force-calcium data is not predictive of cellular phenotype by itself. For example, a reduction in calcium sensitivity of myofilaments can be compensated for by an increase in calcium transient. In such a case there will be no apparent change in contractile function at the cellular level. Similarly, an increase in calcium sensitivity of myofilaments can be offset by defects in mechano-sensing. In fact these compensatory changes are integral to the process of cardiac remodeling leading to DCM. Further, as is evident in many DCM mutants (see Table 1), change in calcium sensitivity is not the only predisposing factor in DCM and the acto-myosin ATPase activity should also be modeled in. Therefore, it is important to factor in the contractile phenotype from an intact cell and incorporate dynamic coupling in a model between thin-thick myofilaments, passive tension, calcium homeostasis, and mechano-transduction, at the least to be considered a cellular level model.

Given the gaps in our knowledge of kinetic rates during molecular transitions and variations in experimental data collated from numerous studies, the mathematical modeler is required to carefully choose explicit Markov states within an appropriate framework, and to standardize technical variates of species, temperature, and molecular state in order to glean meaningful insights from any multi-scale computational study. It is known that various rodent species (mouse, rat, guinea pig) express variable isoforms of key myofilament proteins under normal conditions, for e.g., mouse and rat express α-myosin heavy chain (MHC) isoform (Rundell et al., 2005), whereas, guinea pigs express β-MHC under basal conditions (van der Velden et al., 1998). Given that α-MHC is at least three times faster than β-MHC, this difference in basal conditions in rodents is sufficient to alter the rate of XB cycling significantly (Rundell et al., 2005). Isoform switches in myofilament proteins are known to occur in patho-physiological conditions as well (Nakao et al., 1997). Similar changes in protein isoform expression and kinetics have been reported for other myofilament proteins (de Tombe and Solaro, 2000). These changes impact the myofilament properties of calcium sensitivity, cooperativity and maximal force output significantly. Additionally, increased temperature is known to have a significant effect on increasing calcium sensitivity of myofilaments and increasing the maximal developed contractile force under steady-state conditions in intact and skinned ventricular tissue/cells from various species (Harrison and Bers, 1989, 1990; de Tombe and Stienen, 2007). This is likely due to alterations in molecular kinetics of state transitions of myofilament proteins with temperature. Also, kinetics of state-transitions can vary significantly in isolated molecular states vs. integrated systems, as elucidated in an earlier study where the off-rates of calcium binding were studied in depth from isolated cTnC molecule to a structurally integrated myofilament preparation (Davis and Tikunova, 2008). These biophysical variates must be accounted for during longitudinal scaling from molecular states to myofilament states, as failing to do so can potentially exaggerate or mask the magnitude of, and can even potentially alter the direction of, steady-state myofilament properties as reported for a mutation. It is imperative to note that this is not always possible due to paucity of experimental data, still, careful consideration must be given to these variates in order to arrive at meaningful analysis from *in-silico* studies and to achieve good quantitative agreement with experimental data.

## Insights and challenges while scaling from thin filament mechanics to thick filament mechanics

Results of Markov models of cooperative myofilament activation with nearest neighbor interactions just described suggest that a meaningful prediction toward patho-physiological outcome of genetic mutations is possible *in-silico* (Dewan et al., 2016; Sewanan et al., 2016). While this, of course, does not mean that the models are entirely correct in their predictions, as the whole system is not explicitly represented; it does suggest that the key myofilament properties are adequately represented to recapitulate fairly complex phenomena. These Markov models adequately describe the process of thin filament activation, such that the myofilament contractile response is mediated by a prescribed length-clamp and/or calcium-clamp at any given point of time. However, under physiological conditions, myofilament activation and XB cycling are temporally modulated by cyclical variation in length, calcium, and loading conditions during a contraction-relaxation cycle (ter Keurs et al., 1988). Modeling thick-thin myofilament effects into a dynamic model of contraction, wherein length-dependence and load-dependence of the myofilaments have been incorporated, will allow us to further gain quantitative insights in the shortening response of the contractile machinery and the work done by myofilaments. This is imperative to scaling the effects of the identified DCM mutant from genotype to phenotype.

The length-dependence of myofilament activation and the load-dependence of the rate of myofilament shortening, are tightly regulated and complex properties of the healthy myocardium that are highly dependent on the spatio-temporal arrangement of the myofilaments. In a simplistic view of our understanding of cardiac biomechanics, length dependence of myofilament activation is modulated by the degree of overlap between thick and thin filaments and calcium sensitivity of myofilaments (Huxley and Hanson, 1954; Huxley and Niedergerke, 1954; Dobesh et al., 2002; de Tombe et al., 2010). On the other hand, load-dependence of rate of myofilament shortening is modulated by number of XBs in the strongly bound state, distance between the thick and thin filaments, myosin isoform, degree of thick-thin filament cooperativity, rate of XB cycling, and stiffness of XBs (Edman, 1979; Hunter et al., 1998; McDonald et al., 1998; Herron et al., 2001; Janssen et al., 2002; Konhilas et al., 2002). Ultimately, the twitch dynamics of the myofilaments are the key determinants of the cardiac pump output during various phases of the cardiac cycle (Moss and Buck, 2011). In reality, we are yet to fully comprehend the molecular basis of these dynamic emergent myofilament properties and their modulators. An explicit PDE model that simulates the myofilament contraction-relaxation cycle while recapitulating length-dependent myofilament activation and force-velocity relationships is yet to be described to the best of our knowledge. However, phenomenological models based on empirical relationships have been described to quantitatively recapitulate these myofilament properties (Hunter et al., 1998; Lumens et al., 2009; Kerckhoffs et al., 2010). Phenomenological vs. explicit Huxley-type muscle models of XB cycling have been discussed before (Winters and Stark, 1987).

Computational models of XB cycling dynamics are key toward scaling-up and integrating length dependence and dynamic muscle activation into studies of thin filament DCM proteins. For example, the Rice ODE model of XB cycling accurately mimics length dependence of active/passive forces and Ca^2+^ binding, while simplifying a complex myofilament geometry by keeping track of the overlap fraction between thick and thin filaments (Rice et al., 2008). This model has been expanded in more recent studies to include more complex geometries and metabolic intermediates, and has been incorporated into organ-scale finite element models through solving the sarcomere model at different points in a finite element mesh (Gurev et al., 2010; Tran et al., 2010; Sugiura et al., 2012; Washio et al., 2012). Although, the model traditionally calculates force from a half sarcomere and extrapolates to cell-level force values, it may be possible to explicitly model a full cell with 32 sarcomeres using a Blue Gene supercomputer in order to capture cooperativity of the whole cell (Hussan et al., 2006). Another XB modeling approach more explicitly models the sarcomere geometry by including 3 half thick filaments surrounded hexagonally by 13 half thin filaments, mathematically modeled by a 3D spring array using finite element analysis and governed by Monte Carlo processes (Chase et al., 2004). This model importantly includes filament compliance in its calculations, creating a more biophysically accurate window into the individual proteins making up the myofilament. The Chase model has been used to study specific familial HCM-associated cTnI mutations by converting experimentally gathered Ca^2+^ transient information into altered probability of XB activation (Kataoka et al., 2007). However, this study assumes that altered Ca^2+^ binding kinetics are the sole contributor to altered contractility without considering the interaction of mutated cTnI with other thin filament proteins. When searching for XB models that will be able to accurately model DCM sarcomere mutations, mechanistic models are more promising as they allow for direct input of protein-level changes into a larger scale model.

Phenomenological computational models of twitch tension, which do not explicitly translate the molecular behavior of myofilament proteins to the myofilament properties, can still provide invaluable insight in the workings of the myocardium. Therefore, it is critical to underscore the key considerations and challenges to take into account when formulating dynamic models of cardiac twitch. Temperature dependence of myofilament dynamics, loading conditions (preload and afterload), frequency of electrical pacing which determines the calcium load, and biological species, are biophysical experimental variates that significantly alter twitch dynamics (Stull et al., 2002). Previous studies have reported frequency and temperature dependent changes in contraction-relaxation dynamics and maximal twitch force, based on phase-plane analysis of dynamic force-calcium relationships from intact rat myocardium (Janssen et al., 2002). Briefly, increasing frequency of stimulation resulted in a increased developed force, enhanced myofilament responsiveness to calcium and abbreviated twitch duration; increasing temperature from (22 to 37°C), while maintaining stimulation frequency and preload, resulted in biphasic effects - between 22 and 30°C, developed force did not decrease despite abbreviated twitch duration; between 30 and 37°C, a steep decline in developed twitch force was observed. This would suggest independent temperature dependence on myofilament activation and relaxation kinetics. An earlier study elegantly measured the relationship between cardiac work output and external load in single rat cardiomyocytes (Nishimura et al., 2004). They reported work output of cardiomyocytes under a range of loading conditions ranging from isometric, unloaded and physiologically loaded conditions. Alterations in loading conditions affect XB cycling rates and force-velocity relations. Factoring in the effect of loading conditions, imposed on the myocyte during experimental study from which parameters are being collated, is crucial to correct interpretation of modeling results and for scaling the results to the organ level. Lastly, different experimental studies report data from different biological species, each of which expresses different myofilament protein isoforms that can independently alter twitch dynamics (Clark et al., 1982; Tøndel et al., 2015). It is important that careful consideration be given to these variables as models are parameterized.

## Summary, perspectives and conclusions

**Figure 3:**
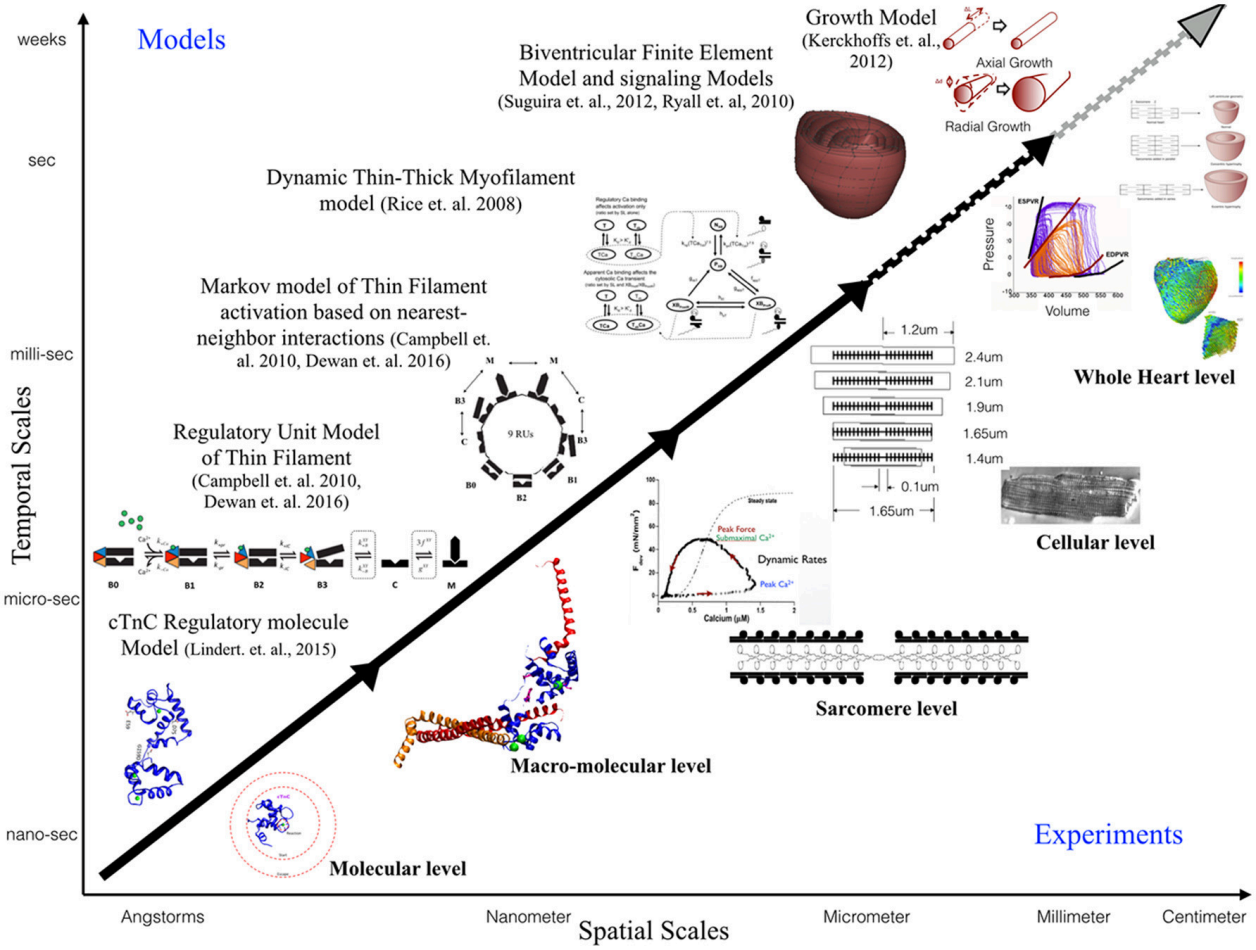
**Schematic illustrating the scope of multi-scale modeling techniques, panning spatial and temporal scales, to complement experimental data at all biological scales**. Representative examples of modeling frameworks from molecular to cellular (solid black arrow—focus of this review) to whole heart level (dotted black arrow) and growth modeling (dotted gray arrow) are shown.

This review is focused toward demonstrating the feasibility and applicability of multi-scale modeling to gain mechanistic insights into role of known genetic DCM mutations in contractile proteins (Figure 3). We discuss some of the insights that can be gained from computational models of cardiac biomechanics when scaling from molecular states to cellular level states. We have described the considerations and challenges that need to be accounted for, when computationally modeling the mechanical effects of sarcomeric mutations of DCM from genotype to cellular phenotype. In particular, we discussed the difficulties in standardizing parameters for a multi-scale modeling study from varied experimental data sets. An important aspect that requires careful consideration and work in modeling studies is coupling of calcium homeostasis and mechano-transduction mechanisms to myofilament mechanics. Sarcomere generated force cannot predict the organ level phenotype in entirety if upstream and downstream regulatory mechanisms in the cell are not coupled to it. In order to comprehensively translate to the cellular scale, coupling of models of active tension to models of (a) passive tension mediated by Titin, (b) upstream calcium homeostasis mechanisms mediating CICR, (c) cytoskeletal proteins and z-disc proteins that sense and transmit force signals, (d) ATP, ROS and energy regulation by mitochondria, (e) insulin signaling, (f) beta-adrenergic signaling pathways, and (g) signaling networks mediating nuclear transcription response toward sarcomere addition should be considered. However, with added complexity in models the degree of ambiguity in results also increases which may prove futile. It is important to note that DCM is a disease characterized by an organ level phenotype, so in order to understand the complex cascade that can cause a point mutation in a contractile protein to lead to growth and remodeling of the heart as a whole it is necessary to scale current molecular and cellular level models to finite element models of the heart with realistic geometries. For more information on whole heart modeling and its potential, detailed reviews and modeling frameworks exist (Campbell and McCulloch, 2011; Trayanova, 2011; Sugiura et al., 2012; Zhang et al., 2016). Of note is the recent study reporting phenomenological modeling of the tension integral of the cardiomyocyte twitch as the predictor for DCM and HCM phenotype in cardiomyopathies (Davis et al., 2016). Furthermore, given that familial DCM manifests clinically over-time it is pertinent to simulate pathological cardiac remodeling leading to DCM. Growth modeling frameworks utilizing stress or strain as the growth stimulus for sarcomere addition at the whole-heart level coupled with cardiac mechanics have been recently reviewed and present with a promising avenue to scale-up (Göktepe et al., 2010; Kerckhoffs et al., 2012). In addition, mechanistic frameworks for modeling biochemical signaling networks that have previously been formulated (Zeigler et al., 2016), such as for PKA mediated beta-adrenergic signaling (Ryall et al., 2012), will be useful tools as cardiac signaling in DCM is being studied based on RNAseq and phosphoprotemics (Burke et al., 2016; Kuzmanov et al., 2016). Formulating explicit models of molecular behavior and scaling them longitudinally to cellular levels is an extremely challenging task and perhaps not without its own pitfalls. Nonetheless, *in-silico* modeling of cardiac biomechanics, in parallel with the experimental studies, is a powerful set of tools that can be applied toward integrative and quantitative understanding of DCM.

## Author contributions

SD took a lead on the project, conceptualized the idea and wrote the article. KM contributed toward conceptualizing and writing the article. MR and AM supervised the project.

## Disclosure

AM is a co-founder, scientific advisor and equity-holders of Insilicomed, Inc., a licensee of UC San Diego software that was not used in this research. Insilicomed, Inc. had no involvement at all in design, performance, analysis or funding of the present study. This relationship has been disclosed to, reviewed, and approved by the University of California San Diego in accordance with its conflict of interest policies. The other authors have no relationships to disclose.

### Conflict of interest statement

The authors declare that the research was conducted in the absence of any commercial or financial relationships that could be construed as a potential conflict of interest. The reviewer JRP and handling Editor declared their shared affiliation, and the handling Editor states that the process nevertheless met the standards of a fair and objective review.
